# Nonrigid Medical Image Registration Using an Information Theoretic Measure Based on Arimoto Entropy with Gradient Distributions

**DOI:** 10.3390/e21020189

**Published:** 2019-02-18

**Authors:** Bicao Li, Huazhong Shu, Zhoufeng Liu, Zhuhong Shao, Chunlei Li, Min Huang, Jie Huang

**Affiliations:** 1 School of Electronic and Information Engineering, Zhongyuan University of Technology, Zhengzhou 450007, China; 2 Laboratory of Image Science and Technology, School of Computer Science and Engineering, Southeast University, Nanjing 210096, China; 3 College of Information Engineering, Capital Normal University, Beijing 100048, China; 4 School of Computer and Communication Engineering, Zhengzhou University of Light Industry, Zhengzhou 450002, China

**Keywords:** Arimoto entropy, free-form deformations, normalized divergence measure, gradient distributions, nonextensive entropy, non-rigid registration

## Abstract

This paper introduces a new nonrigid registration approach for medical images applying an information theoretic measure based on Arimoto entropy with gradient distributions. A normalized dissimilarity measure based on Arimoto entropy is presented, which is employed to measure the independence between two images. In addition, a regularization term is integrated into the cost function to obtain the smooth elastic deformation. To take the spatial information between voxels into account, the distance of gradient distributions is constructed. The goal of nonrigid alignment is to find the optimal solution of a cost function including a dissimilarity measure, a regularization term, and a distance term between the gradient distributions of two images to be registered, which would achieve a minimum value when two misaligned images are perfectly registered using limited-memory Broyden–Fletcher–Goldfarb–Shanno (L-BFGS) optimization scheme. To evaluate the test results of our presented algorithm in non-rigid medical image registration, experiments on simulated three-dimension (3D) brain magnetic resonance imaging (MR) images, real 3D thoracic computed tomography (CT) volumes and 3D cardiac CT volumes were carried out on *elastix* package. Comparison studies including mutual information (MI) and the approach without considering spatial information were conducted. These results demonstrate a slight improvement in accuracy of non-rigid registration.

## 1. Introduction

Volume registration is an essential task of image processing, especially in medical field, such as aiding diagnosis, surgical applications, and image-guided radiation therapy [1,2]. The images to be registered are generally obtained at different times and from different imaging sensors, namely, multi-modality imaging. Different medical imaging modalities could provide various and complementary information. For instance, CT and MRI display the anatomic structures of an organ, while positron emission tomography (PET) and single photon emission computed tomography (SPECT) provide the functional and metabolic information. Therefore, in clinic, these multi-modal volumes are often registered and fused together, in this way, much complementary information derived from different modalities are supplied with the physicians to improve the diagnosis accuracy and assessment efficiency of lesion progression.

Image registration is related by the process to find the optimal mapping function between two images to be aligned [3]. In recent years, the registration algorithms using the similarity based on information theory have been attracted more and more attention in medical image registration, among which maximization of MI was early reported for registration of medical images from different modalities by Collignon et al. [4], Maes et al. [5], Wells et al. [6]. Studholme et al. [7] studied a normalized similarity measure called normalized MI (NMI) to tackle the problem of changing field of view (FOV). MI and NMI are both estimated by the probability distributions of images to be registered. Besides, the concept of cumulative probability distributions was introduced into image registration, and cumulative residual entropy (CRE) was investigated in [8]. Additionally, the relations between CRE and Shannon entropy were ulteriorly researched in [9], and a comparison with MI was reported in [10]. In this new measure, cumulative density functions (CDF) instead of probability density functions (PDF) were adopted to calculate values of the similarity measure, which illustrates a good robustness to noise.

The aforementioned measures based on information theory—such as MI, NMI, and CRE—are constructed by Shannon entropy. Nonetheless, the additivity of Shannon entropy signifies that it is an extensive entropy. However, Antolin et al. [11] pointed out that the extensive entropy does not consider the correlation of two variables. Consequently, they presented a similarity measure exploiting Tsallis entropy. Subsequently, this new divergence was employed to construct a non-rigid registration model for medical images [12,13].

Illuminated by the reference [11], a divergence measure based on Arimoto entropy was presented called the Jensen–Arimoto divergence (JAD) [14]. In [15], some properties, such as the concavity of Arimoto entropy and the boundedness of JAD, have been further investigated. However, the registration method based on JAD does not take the spatial information between voxels into account, which is of significance to medical image registration. This paper aims to present a novel nonrigid registration method of medical images adopting a normalized measure based on Arimoto entropy and gradient distributions. Firstly, the properties of Arimoto entropy and JAD are analyzed, and a distance of gradient distributions is constructed. Secondly, a nonrigid deformation model is chosen and the registration process is formulated by an optimization procedure. In the sequel, the continuous probability distributions are estimated using Parzen window method applying B-splines and the analytical gradient of objective function can be obtained. Finally, the L-BFGS optimization [16] is adopted to obtain the optimal deformation parameters. To assess the performance of our registration framework for medical images, several groups of non-rigid experiments on simulated volumes and real 3D data are implemented.

Our contributions are twofold. Firstly, the related measures based on Shannon entropy do not consider the correlation. Therefore, we present a normalized measure based on Arimoto entropy, a non-extensive entropy, as the dissimilarity measure. Secondly, in the existing measures, such as MI, NMI and JAD, the intensity values are directly exploited to calculate the similarity measure, while the spatial information has been not considered. To take the spatial information between voxels into account, a distance term of gradient distributions is constructed and incorporated into the objective function.

The rest of this work is arranged as follows. In Section 2, the knowledge of information theory was firstly reviewed, and then introduce the Arimoto entropy and JAD measure, constructing gradient distribution distance. We formulate the model of nonrigid registration and give the detailed description of the registration method in Section 3 adopting a normalized measure based on Arimoto entropy with gradient distribution. Section 4 demonstrates the nonrigid test results on 3D MR volumes and real 3D clinical datasets, with the compared results to other registration algorithms illustrated. Finally, we provide the conclusions and perspectives in Section 5.

## 2. Preliminaries

For this section, we briefly review the theoretical concept of information theory, and then introduce the Arimoto entropy and JAD, along with studying their properties. In addition, the gradient distributions of reference image and float image are constructed and a distance of them is derived.

### 2.1. Shannon Entropy and Mutual Information

For an arbitrary random variable *X*(*x*_1_, *x*_2_,…, *x_N_*), with its probability distributions *p*(*x*_1_, *x*_2_,…, *x_N_*), the Shannon entropy of *X* is used to measure the amount of average uncertainty included in this random variable,

(1) $$H(X)=-\sum_{i=1}^{N}p(x_{i})\log p(x_{i})$$

which is also employed to measure the account of information provided by this random variable. Then, considering another random variable *Y*, *H*(*X*|*Y*) is remarked as the conditional entropy of *X* when *Y* is known. The reduction in uncertainty due to *Y* is called the MI. MI of *X* and *Y* is defined by

(2) $$I(X,Y)=H\left(X\right)-H\left(X|Y\right)=\sum_{x}\sum_{y}p(x,y)\log\frac{p(x,y)}{p(x)\cdot p(y)}$$

where *p*(*x*) and *p*(*y*) denote marginal probability of two random variables, as well as *p*(*x*, *y*) being the joint distribution of them. MI is applied as a measure of the dependence between *X* and *Y*. MI is symmetric in *X* and *Y* and always nonnegative [17].

### 2.2. Arimoto Entropy

Arimoto [18] introduced a generalized form of Shannon entropy, Arimoto entropy is defined by

(3) $$A_{\alpha }(X)=\frac{\alpha }{\alpha -1}\left[1-{\left(\sum_{i=1}^{N}p_{i}^{\alpha }\right)}^{\frac{1}{\alpha }}\right]\;\alpha >0,\alpha \neq 1$$

Boekee et al. [19] investigated some significant properties of Arimoto entropy, here, we only exhibit several useful properties as follows.

Non-negativity:

(4) $$A_{\alpha }(X)\geq 0\;\alpha >0,\alpha \neq 1$$

Pseudo-additivity:

(5) $$A_{\alpha }(X,Y)=A_{\alpha }(X)+A_{\alpha }(Y)-\frac{\alpha -1}{\alpha }A_{\alpha }(X)A_{\alpha }(Y)\;\alpha >0,\alpha \neq 1$$

Concavity:

(6) $$A_{\alpha }(tX_{1}+(1-t)X_{2})\geq tA_{\alpha }(X_{1})+(1-t)A_{\alpha }(X_{2})\;t\in (0,1),\alpha >0,\alpha \neq 1$$

Symmetry:

(7) $$A_{\alpha }\left(\cdots ,p_{i},\cdots p_{j}\cdots \right)=A_{\alpha }\left(\cdots ,p_{j},\cdots p_{i}\cdots \right)$$

Upper bound:

(8) $$A_{\alpha }\left(p_{1},p_{2},\cdots ,p_{N}\right)\leq A_{\alpha }\left(\frac{1}{N},\frac{1}{N},\cdots ,\frac{1}{N}\right)=\frac{\alpha }{\alpha -1}\left[1-N^{\frac{1-\alpha }{\alpha }}\right]$$

See [19] for the detailed proof of these properties. Property one ensures that Arimoto entropy is non-negative. Its pseudo-addivity illustrates that Arimoto entropy accounts for a non-extensive entropy. The parameter α in (3) accounts for the degree of non-extensivity.

### 2.3. Jensen Arimoto Divergence

For a random variable X, and $P(p_{1},p_{2},\ldots ,p_{N})$ is probability distributions on *X*. JAD is defined to be [15]

(9) $$JA_{\alpha }(p_{1},p_{2},\ldots ,p_{N})=A_{\alpha }\left(\sum_{i=1}^{N}\omega_{i}p_{i}\right)-\sum_{i=1}^{N}\omega_{i}A_{\alpha }(p_{i})\;\alpha >0,\;\alpha \neq 1$$

where $A_{\alpha }(\cdot )$ denotes Arimoto entropy and $\omega_{i}$ is a weighted vector to constrain $\omega_{i}\geq 0$ and $\sum_{i=1}^{N}\omega_{i}=1$. In the following, we review the properties of JAD.

**Proposition** **1.** *JAD has these properties of non-negativity, symmetry. Also, JAD is identical to 0 when and only when all of the probability distributions are the same as each other. The proof has been reported in [15].*

Li et al. pointed that a distance must fulfill four requirements [20]. JAD does not meet the triangle inequality, so JAD is not a true distance metric. Nonetheless, JAD can still be adopted to measure the disparity among the probability distributions of two random variables.

**Proposition** **2.** *The JA divergence has the maximum when **p**_1_, **p**_2_,…, **p**_N_ are degenerate distributions, where $p_{i}=\delta_{ij}=1$ when i = j and 0 otherwise. The proof had been provided in [21], and the maximum equals to $A_{\alpha }(\omega )$.*

According to Proposition 1 and 2, it is obviously observed that JAD is bounded, $0\leq JA_{\alpha }(p_{1},p_{2},\ldots ,p_{N})\leq A_{\alpha }(\omega )$.

### 2.4. Gradient Distributions Distance

Given the reference image *R* and float image *F*, ∇*F*, and ∇*R* represent the gradients of *F* and *R*, along with *q*(∇*F*) and *p*(∇*R*) representing the gradient distributions of *F* and *R*. Kullback–Leibler divergence (KLD) can be used to calculate the distance of *q*(∇*F*) and *p*(∇*R*) as

(10) $$KLD\left(q||p\right)=\sum_{x}q\left(\nabla F\left(x\right)\right)\log\frac{q\left(\nabla F\left(x\right)\right)}{p\left(\nabla R\left(x\right)\right)}$$

where x denotes any point of image gradient, x = [*x*, *y*, *z*]^T^. KLD is also known as relative entropy, measuring the diversity of two probability distributions. In (10), we use the convention that 0·log(0/0) = 0. In other word, when *R* and *F* are completely registered, KLD between gradient distributions defined in (10) achieves the minimum value. Considering the spatial transformation *T_μ_*, *μ* denoted by the parameters of transformation model, the gradient distribution distance of *F* and the transformed *F* is given by

(11) $$KLD\left(\nabla F\left(T_{\mu }\left(x\right)\right)||\nabla R\left(x\right)\right)=\sum_{d}\sum_{x}q\left(\nabla F_{d}\left(T_{\mu }\left(x\right)\right)\right)\log\frac{q\left(\nabla F_{d}\left(T_{\mu }\left(x\right)\right)\right)}{p\left(\nabla R_{d}\left(x\right)\right)}$$

where ∇*F_d_*(*T_μ_*(x)) and ∇*R_d_*(x) represent the gradients of transformed float image and reference image, and *d* is the dimension of the images to be registered. Subsequently, the Parzen method is employed to estimate the gradient distributions. 

Denote *β*^(0)^ and *β*^(3)^ by zero-order and three-order B-spline, respectively. In the process of image registration, the transformation parameters *μ* does not affect the reference image, the gradient of reference image is also constant in registration process. Consequently, the gradient of reference image can be calculated before registration to improve the implementation efficiency. In addition, a zero-order B-spline is exploited to estimate the gradient distributions ∇*R_d_*(x) of reference image *R*(x), with the probability density function of ∇*R_d_*(x) defined by

(12) $$p\left(\nabla R_{d}\left(x\right)\right)={\tilde{p}}_{d}(r_{i})=\frac{1}{V}\sum_{x\in \Omega }\beta^{\left(0\right)}\left(r_{i}-\frac{\nabla R_{d}(x)-\nabla R_{d}^{0}}{\Delta b_{R}}\right)$$

where Ω is volume domain to estimate probabilities. *V* denotes the number of voxels of Ω domain, as well as *d* being the image dimension. *r_i_* represents intensity levels of ∇*R_d_*(x), and ‘∇’ is the gradient operator. The three-order B-spline is adopted to compute the gradient distributions ∇*F_d_*(*T_μ_*(x)) of transformed float image, with the probability density function of ∇*F_d_*(*T_μ_*(x)) shown as

(13) $$q\left(\nabla F_{d}\left(T_{\mu }\left(x\right)\right)\right)={\tilde{q}}_{d}(f_{j})=\frac{1}{V}\sum_{x\in \Omega }\beta^{\left(3\right)}\left(f_{j}-\frac{\nabla F_{d}(T_{\mu }(x))-\nabla F_{d}^{0}}{\Delta b_{F}}\right)$$

In Equations (12) and (13), Δ*b_R_* and Δ*b_F_* are the widths of bins. $\nabla R_{d}^{0}$ and $\nabla F_{d}^{0}$ is the minimum values in two images, and *f_j_* represents intensity levels of ∇*F_d_*(*T_μ_*(x)). Substituting (12) and (13) into (11), we can obtain the following formula.

(14) $$\begin{array}{ll} KLD\left(\nabla F\left(T_{\mu }\left(x\right)\right)||\nabla R\left(x\right)\right)= & \sum_{d}\sum_{x}{\tilde{q}}_{d}(f_{j})\log\frac{{\tilde{q}}_{d}(f_{j})}{{\tilde{p}}_{d}(r_{i})}\\ & \;=\sum_{d}\left[H\left({\tilde{q}}_{d}\right)-\sum_{x}{\tilde{q}}_{d}(f_{j})\log{\tilde{p}}_{d}(r_{i})\right] \end{array}$$

where *H* is the Shannon entropy. In this paper, KLD of gradient distributions will be applied as a distance term in medical image registration, regularizing that the gradient distribution of float image is similar to the gradient distribution of reference image.

## 3. Description of Proposed Nonrigid Registration Method

**Figure 1:**
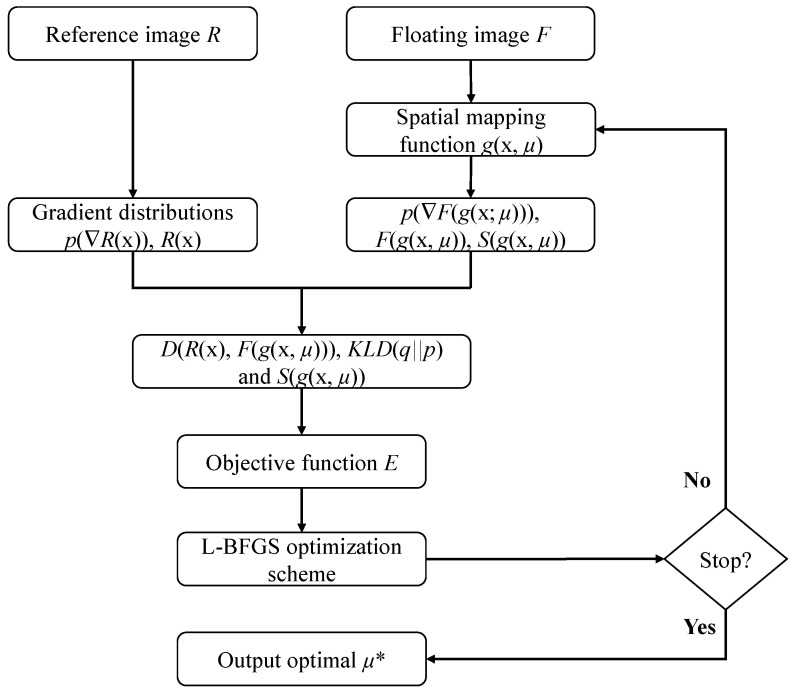
Block diagram of our registration algorithm.

The details of the registration method using a normalized information theoretic measure based on Arimoto entropy with gradient distributions is described. Firstly, an appropriate transformation model needs to be selected, where the registration criteria (objective function or cost function) and optimization algorithm is applied to optimize the criteria. Finally, the images to be registered are aligned using an optimal solution obtained by the optimization scheme. Figure 1 displayed the block diagram of our nonrigid registration framework.

### 3.1. Formulation

**Figure 2:**
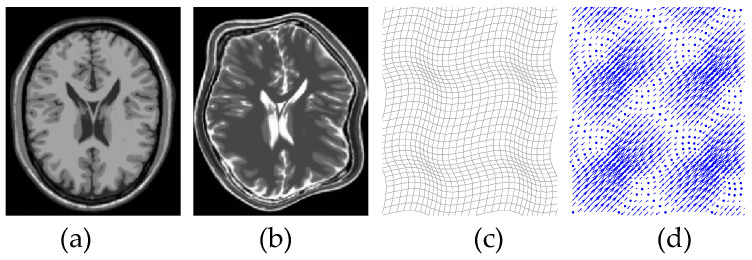
(**a**) MR T1 image; (**b**) MR T2 image; (**c**) deformation field; (**d**) deformation vector.

Figure 2a,b depict the corresponding planes in two 3D MR images, which account for T1-weighted MR and T2-weighted MR. Non-rigid registration is formulated as the process of searching for the optimal spatial deformation function of reference image *R* and float image *F* as

(15) y=g(x;μ)

where *g*(x; *μ*) is the deformation function, *μ* denotes the vector of deformation parameters, with x and y being the coordinates of arbitrary point in *R* and *F*, respectively. We can formulate the non-rigid registration of *F* to *R* as

(16) $$T^{*}=\underset{\mu }{\arg\min}D\left(F\left(x\right)\circ g\left(x;\mu \right),R\left(x\right)\right)=\underset{\mu }{\arg\min}D\left(F\left(g\left(x;\mu \right)\right),R\left(x\right)\right)$$

where *D* represents a dissimilarity measure that can achieves its minimum in registration of *R*(x) and *F*(*g*(x; *μ*)). 

However, the process of image registration is an ill-posed issue, and a penalty term need to be incorporated to obtain a smooth transformation. Considering the regularization term, the cost function *E* is expressed by

(17) E=D(F(g(x;μ)),R(x))+λS(g(x;μ))

### 3.2. Transformation Model

Clinically, there exist some large deformations between medical images. Therefore, a nonrigid transformation is generally employed to deal with organ or tissue deformation. Figure 2c,d display the deformation fields and vectors of the nonrigid transformation between Figure 2a,b. The free-form deformations (FFD) model can preferably described local deformations between medical images. Therefore, cubic B-splines are employed to constructed FFD model [22] to simulate this elastic deformation.

In a 3D image, Φ is a mesh with the size of *n_x_* × *n_y_* × *n_z_*, as well as the control points [*ω_i_*, *ω_j_*, *ω_k_*]*^T^*, and *δ* is the size of spacing. Then, 3D deformation function of x = [*x*, *y*, *z*]*^T^* could be defined as

(18) $$g\left(x;\mu \right)=\sum_{ijk}\mu_{ijk}\beta^{\left(3\right)}\left(\frac{x-\omega_{i}}{\delta }\right)\beta^{\left(3\right)}\left(\frac{y-\omega_{j}}{\delta }\right)\beta^{\left(3\right)}\left(\frac{z-\omega_{k}}{\delta }\right)$$

where *μ_ijk_* is the vector of deformation coefficients, and *β*^(3)^ is the third-order B spine function.

### 3.3. Registration Criteria

The dissimilarity measure *D* is the most significant part of objective functions, which is used to measure the difference between two images to be registered. When *D* achieve the minimum value, the similarity of two volumes is maximized, resulting in the two volumes completely registered. We have employed JAD as similarity measure to register medical images in the presence of non-rigid transformation, in which a negative sign is assigned to the JAD to construct the dissimilarity measure [15]. In this paper, according to *Proposition 2* in Section 2.3, a normalized dissimilarity measure based on JAD is introduced. Its definition is given as

(19) $$D\left(F(g(x;\mu )),R(x)\right)=1-\frac{JA_{\alpha }\left(F(g(x;\mu )),R(x)\right)}{A_{\alpha }\left(\omega \right)}$$

In [15], JAD is expressed by

(20) $$\begin{array}{ll} JA_{\alpha }\left(F(g(x;\mu )),R(x)\right) & =\;\frac{\alpha }{1-\alpha }\{{\left[{\sum_{j=1}^{M}\left[\sum_{i=1}^{M}p(r_{i})p(f_{j}|r_{i})\right]}^{\alpha }\right]}^{\frac{1}{\alpha }}\;-\sum_{i=1}^{M}p(r_{i}){\left[\sum_{j=1}^{M}p{\left(f_{j}|r_{i}\right)}^{\alpha }\right]}^{\frac{1}{\alpha }}\}\\ & =\frac{\alpha }{1-\alpha }\left\{{\left[\sum_{j=1}^{M}p{\left(f_{j}\right)}^{\alpha }\right]}^{\frac{1}{\alpha }}-\sum_{i=1}^{M}{\left[\sum_{j=1}^{M}p{\left(r_{i},f_{j}\right)}^{\alpha }\right]}^{\frac{1}{\alpha }}\right\} \end{array}$$

where *f* = (*f*_1_, *f*_2_, …, *f_M_*) and *r* = (*r*_1_, *r*_2_, …, *r_M_*) are the intensity values in *F*(*g*(x; *μ*)) and *R*(x). Also, *M* is bins number. Also, *p*(*f_j_*|*r_i_*) is the conditional probability. JAD and *A_α_*(*ω*) are substituted into (19). In consequence, the dissimilarity measure *D* in (19) is rewritten as

(21) $$D\left(F(g(x;\mu )),R(x)\right)=1-\left\{\sum_{i=1}^{M}{\left[\sum_{j=1}^{M}p{\left(r_{i},f_{j}\right)}^{\alpha }\right]}^{\frac{1}{\alpha }}-{\left[\sum_{j=1}^{M}p{\left(f_{j}\right)}^{\alpha }\right]}^{\frac{1}{\alpha }}\right\}/\left(1-M^{\frac{1-\alpha }{\alpha }}\right)$$

where *M* is the number of bins. To address the problem of nonrigid registration, the smooth deformation needs to be acquired by regularizing the deformation model. Incorporated with the regularization term, the objective function *E* is rewritten by

(22) E(F(g(x;μ)),R(x))=D(F(g(x;μ)),R(x))+λS(g(x;μ))

where *D* is the dissimilarity measure defined in (21), and *S* is the regularization term, with its expression given as follows.

(23) $$\begin{array}{ll} S(g\left(x;\mu \right)) & =\frac{1}{V}\int_{0}^{X}\int_{0}^{Y}\int_{0}^{Z}[{\left(\frac{\partial^{2}g\left(x;\mu \right)}{\partial x^{2}}\right)}^{2}+{\left(\frac{\partial^{2}g\left(x;\mu \right)}{\partial y^{2}}\right)}^{2}+{\left(\frac{\partial^{2}g\left(x;\mu \right)}{\partial z^{2}}\right)}^{2}\\ & +2{\left(\frac{\partial^{2}g\left(x;\mu \right)}{\partial x\partial y}\right)}^{2}+2{\left(\frac{\partial^{2}g\left(x;\mu \right)}{\partial x\partial z}\right)}^{2}+2{\left(\frac{\partial^{2}g\left(x;\mu \right)}{\partial y\partial z}\right)}^{2}]dxdydz, \end{array}$$

However, objective function shown in (22) does not take into account the spatial information between voxels. To deal with the issue, the distance between two gradient distributions *q*(∇*F*(*g*(x; *μ*))) and *p*(∇*R*(x)) displayed in (14) is introduced to (22). As a result, the nonrigid registration process is expressed by

(24) $$\begin{array}{ll} \mu^{*} & =\underset{\mu }{\arg\min}E\left(F(g(x;\mu )),R(x)\right)\;\\ & =\underset{\mu }{\arg\min}\{D\left(F(g(x;\mu )),R(x)\right)+\lambda_{1}S(g(x;\mu ))+\lambda_{2}KLD\left(\nabla F\left(g\left(x;\mu \right)\right)||\nabla R\left(x\right)\right)\} \end{array}$$

where KLD represents gradient distribution distance, as well as *λ*_1_ and *λ*_2_ being weight parameters, balancing the tradeoff among a dissimilarity measure *D*, a regularization *S*, and a distance term KLD.

### 3.4. Optimization

Newton–Raphson algorithm has been widely exploited, in which second-order derivatives can show better convergence [23] compared with these strategies based on first-order gradient. L-BFGS [24] does not calculate second-order information. Thus, a high computation efficiency can be achieved. A second-order Taylor approximation [16] of *E* with respect to *μ* is given as

(25) $$E(\mu +\Delta \mu )\approx E(\mu )+\Delta \mu^{T}\cdot \nabla E(\mu )+\frac{1}{2}\Delta \mu^{T}\cdot \nabla^{2}E(\mu )\cdot \Delta \mu ,$$

where Δ*μ* is the increment of *μ*, ∇ is gradient operation. The deformation parameter *μ* of the L-BFGS optimization algorithm is updated as

(26) $$\mu^{\left(k+1\right)}=\mu^{\left(k\right)}-{\left(H^{\left(k\right)}\right)}^{-1}\cdot \nabla E(\mu^{\left(k\right)}),$$

In the sequel, the derivative of objective function *E* with respect to *μ* need to computed.

(27) $$\frac{\partial E}{\partial \mu }=\left[\frac{\partial E}{\partial \mu_{1}},\frac{\partial E}{\partial \mu_{2}},\cdot \cdot \cdot ,\frac{\partial E}{\partial \mu_{n}}\right],$$

The pseudo code of our registration approach is displayed in Algorithm 1.

To solve the optimization process, we need calculate the analytical gradient of the objective function *E*. Traditionally, the probability distributions expressed in (21) was not continuous. Hence, the continuous probability density function (pdf) needs to be estimated by Parzen-window method. The continuous marginal and joint pdfs of two images to be registered have been calculated [15]. The continuous expression of gradient distribution distance has been also provided in Section 2.4. Equalization (18) is substituted into (23), the continuity of smoothness term is acquired. Consequently, the objective function *E* is continuous and its analytical derivative with respect to *μ* can be calculated.

**Algorithm 1.** Nonrigid medical image registration with gradient distributions**Input:** Reference image *R*, floating image *F* **Output:** Optimal deformation parameters μ* Set
*λ*_1_, *λ*_2_, NMAX, α, M, N, *δ*, *ε* **Compute** the gradient of *R*, denote as ∇*R*(x) and gradient distributions *p*(∇*R*(x)) **Initialize** deformation parameters *μ*^(0)^, iteration *k* = 0, *F*(*g*(x; *μ*^(0)^)) = *F*, *E*(*μ*^(0)^) = 0 **While** |*E*(*μ*^(*k* + 1)^) − *E*(*μ*^(*k*)^)|> threshold *ε* or *k* < =NMAX Obtain the deformed float image *F*(*g*(x; *μ*^(*k*+1)^)) and the regularization *S*(*g*(x, *μ*^(*k* + 1)^)) Compute ∇*F*(*g*(x; *μ*^(*k* + 1)^)) and gradient distributions *q*(∇*F*(*g*(x; *μ*^(*k* + 1)^))) Estimate the dissimilarity measure *D* and gradient distributions distance *KLD* Calculate objective function *E*(*μ*^(*k* + 1)^) = *D*(*R*(x), *F*(*g*(x; *μ*^(*k* + 1)^))) + *KLD*(*q*^(*k* + 1)^*||p*) + *S*(*g*(x, *μ*^(*k* + 1)^)) *μ*^(*k* + 1)^ =*μ*^(k)^ − (*H*^(*k*)^) ^−1^·∇*E*(*μ*^(*k*)^) *k* = *k* + 1 **end**

#### Derivative of the Objective Function

The objective function defined in (24) includes dissimilarity measure *D*, a regularization term *S*, and a distance term KLD. The derivative of *D* is deduced as

(28) $$\frac{d\left[D\left(F(g(x;\mu )),R(x)\right)\right]}{d\mu }=-\frac{1}{A_{\alpha }\left(\omega \right)}\frac{d\left[JA_{\alpha }\left(F(g(x;\mu )),R(x)\right)\right]}{d\mu }$$

According to (20), we obtain

(29) $$\frac{d\left[D\left(F(g(x;\mu )),R(x)\right)\right]}{d\mu }=\frac{1}{\left(1-M^{\frac{1-\alpha }{\alpha }}\right)}\cdot \left\{\sum_{i}\sum_{j}Y\frac{\partial \tilde{p}(r_{i},f_{j})}{\partial \mu }\right\}$$

(30) $$Y={\left(\sum_{j}\tilde{p}{\left(f_{j}\right)}^{\alpha }\right)}^{\frac{1}{\alpha }-1}\tilde{p}{\left(f_{j}\right)}^{\alpha -1}-{\left(\sum_{j}\tilde{p}{\left(f_{j}|r_{i}\right)}^{\alpha }\right)}^{\frac{1}{\alpha }-1}\tilde{p}{\left(f_{j}|r_{i}\right)}^{\alpha -1}$$

where $\partial \tilde{p}(f_{j},r_{i})/\partial \mu$ represents derivative of estimated joint probability. The derivative of $\tilde{p(f_{j},r_{i};\mu )}$ is calculated by

(31) $$\begin{array}{ll} \frac{\partial \tilde{p(f_{j},r_{i};\mu )}}{\partial \mu }= & -\frac{1}{N\cdot \Delta b_{F}}\sum_{x\in \Omega }\beta^{\left(0\right)}\left(r_{i}-\frac{R(x)-R^{0}}{\Delta b_{R}}\right)\\ & \;\times {\beta^{\prime }}^{\left(3\right)}\left(f_{j}-\frac{F(g\left(x;\mu \right))-F^{0}}{\Delta b_{F}}\right)\times \left(\frac{\partial F(s)}{\partial s}{|}_{s=g\left(x;\mu \right)}\right)\times \frac{\partial (g\left(x;\mu \right))}{\partial \mu } \end{array}$$

with *β*^(0)^ and *β*′^(3)^ being the zero-order B-Splines and the derivative of the three-order B-Splines, respectively. *R*^0^ and *F*^0^ are the minimal intensities in *R*(x) and *F*(*g*(x; *μ*)), as well as ∂F(t)/∂t being the gradient of the deformed float image *F*(*g*(x; *μ*)), ∂(g(x;μ))/∂μ can be estimated by FFD model. 

To obtain the derivative of the penalty term *S*, we rewrite (23) as

(32) $$S\left(g(x;\mu )\right)=\frac{1}{V}{\sum_{x}\sum_{i,j=1}^{3}\left(\frac{\partial^{2}g(x;\mu )}{\partial x_{i}\partial x_{j}}\right)}^{2}$$

where x represents the points in image region, and *V* denotes the number of pixels. The derivative of *S* has been provided by Staring and Klein [25],

(33) $$\frac{\partial S\left(g(x;\mu )\right)}{\partial \mu }=\frac{1}{V}\sum_{x}\sum_{i,j=1}^{3}2\left(\frac{\partial^{2}g(x;\mu )}{\partial x_{i}\partial x_{j}}\right)\frac{\partial }{\partial \mu }\frac{\partial^{2}g(x;\mu )}{\partial x_{i}\partial x_{j}}$$

where $\partial^{2}g(x;\mu )/\partial x_{i}\partial x_{j}$ denotes Hessian matrix of deformation function *g*(x; *μ*), $\partial \left(\partial^{2}T/\partial x_{i}\partial x_{j}\right)/\partial \mu$ is the Jacobi of Hessian matrix. 

Next, we calculate the derivative of KLD,

(34) $$\frac{d\left[KLD\left(\nabla F\left(g\left(x;\mu \right)\right)||\nabla R\left(x\right)\right)\right]}{d\mu }=\sum_{d}\sum_{x}\left(1+\log\frac{{\tilde{q}}_{d}(f_{j})}{{\tilde{p}}_{d}(r_{i})}\right)\frac{\partial {\tilde{q}}_{d}(f_{j})}{\partial \mu }$$

where ${\tilde{p}}_{d}(r_{i})$ and ${\tilde{q}}_{d}(f_{j})$ represent the gradient distributions of the transforms float image and reference image, respectively. The derivative of ${\tilde{q}}_{d}(f_{j})$ is calculated as

(35) $$\begin{array}{ll} \frac{\partial {\tilde{q}}_{d}(f_{j})}{\partial \mu }=-\frac{1}{V\cdot \Delta b_{F}} & \sum_{x\in \Omega }\;\cdot {\beta^{\prime }}^{\left(3\right)}\left(f_{j}-\frac{\nabla F_{d}(g(x;\mu ))-\nabla F_{d}^{0}}{\Delta b_{F}}\right)\\ & \;\cdot \left(\nabla^{2}F_{d}(s){|}_{s=g(x;\mu )}\right)\cdot \frac{\partial (g(x;\mu ))}{\partial \mu } \end{array}$$

where *β*′^(3)^ is derivative of three-order B-spline, and $\nabla^{2}F_{d}(t)$ represents the second-order gradient of *F*(*g*(x; *μ*)), as well as ∂(g(x;μ))/∂μ being the derivative of deformation function *g*(x; *μ*) with respect to the parameter *μ*. Substituting (12), (13), and (35) into (34), we can obtain the derivative of gradient distribution distance. In the terms of (29), (33), and (34), the derivative of *E* will be easily calculated.

## 4. Experiments and Results

To evaluate the registration method using the normalized JAD with gradient distribution (NJAD-GD), we designed several groups of tests and performed on simulated and real 3D data, respectively. In Section 4.1, the experimental data is depicted, including simulated and real medical images. The non-rigid registration of simulated MR volumes is performed in Section 4.2. The tests on real 3D thoracic CT images and 3D cardiac data are implemented, and the experimental results are shown in Section 4.3 and Section 4.4, respectively. Our nonrigid registration algorithm employing JAD and gradient distributions was implemented in the *elastix* package [26].

### 4.1. Experimental Data

**Figure 3:**
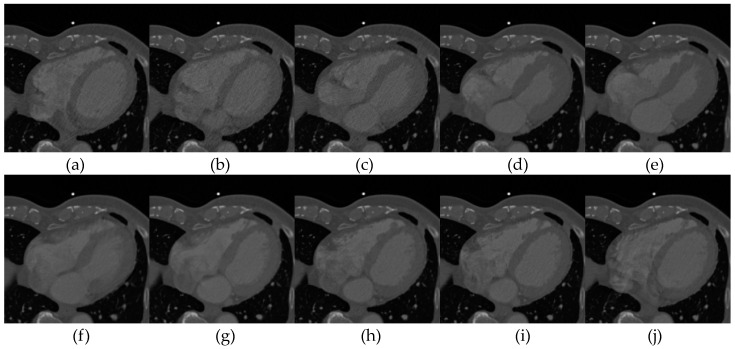
The axis slice of 10 3D cardiac CT images in one 4D sequence. (**a**–**j**) represent the 10 frames acquired from one whole cardiac cycle of one patient.

In this paper, simulated brain MR volumes, thoracic CT volumes and real 3D cardiac CT images were exploited as experimental data. The detailed descriptions of brain MR and 3D thoracic CT images have been reported in [15]. Additionally, non-rigid tests were also performed on twelve 4D cardiac CT sequences acquired from twelve patients. Each of 4D CT sequence consists of 10 3D cardiac CT images, which were obtained from one whole cardiac cycle of one patient. These CT images have 256 × 256 pixels along axial direction. Figure 3 exhibits 10 3D cardiac CT volumes of one 4D CT sequence. It is obviously observed that some elastic deformations are existed between 10 images.

### 4.2. Nonrigid Registration of Simulated Brain Images

Simulated brain volumes were firstly used to design the elastic alignment experiments. Furthermore, we employ a multiresolution hierarchical strategy with three levels to carry out these non-rigid tests. Also, a comparison with JAD without gradient distribution and MI is also reported. 

We selected 60 warping indexes (see parameters *m* of the warping function in [15]), which were yielded randomly from the interval [1,7]. Consequently, 60 float images were produced based on the 60 deformations for each pair of test volumes and 540 nonrigid trials of three pairs of brain MR volumes for NJAD-GD, JAD, and MI algorithms in total. 

To assess quantitatively test results of these trails, we exploit registration error as the evaluation standard. Here, the registration error is defined as the difference of true values that can be calculated by warping indexes and the obtained values by optimization strategy. In the registration trails of brain images, the involved parameters are set as follows: the nonextensive parameter *α* = 1.5, bins M = 16, the number of random samples N = 2000, δ = 20 × 20 × 20. The weighting parameters *λ*_1_ = 0.005 and *λ*_2_ = 0.001 can provide a good tradeoff among three terms: *D*, *S*, and gradient distribution term KLD in the objective function *E*. 

**Figure 4:**
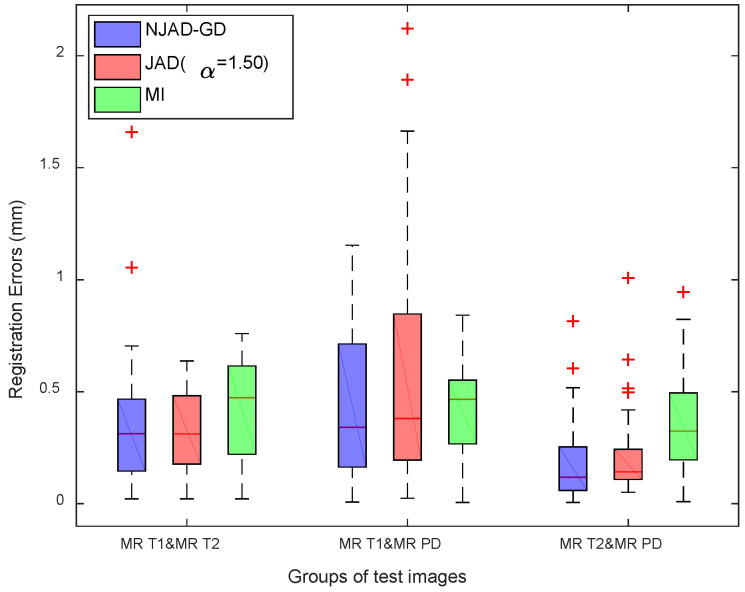
The registration results of the simulated 3D brain MR T1 & MR T2, MR T1 & MR PD, and MR T2 & MR PD volumes using three algorithms. The red color crosses for each box represents these outliers.

Figure 4 shows the test results of all 540 non-rigid registrations. From Figure 4, the NJAD-GD registration algorithm could result in the lower errors of three pairs of test volumes compared to other two approaches.

### 4.3. Experiments of 3D Thoracic CT Images

3D thoracic CT volumes were chosen as the test images to carry out non-rigid registrations. These volumes consist of four 4D sequences, and each of them includes 10 3D volumes. A three-level implementation scheme was still employed to decrease registration accuracy and improve computation efficiency.

We denote the 10 volumes from each 4D sequence by T00-T90, in which the maximal inhalation and maximum exhalation are included, with indicated by T10 and T60, respectively. Then, we designed the following experiments: in each 4D sequence, the T60 frame is applied as reference image and the residual nine frames are chosen as float image, leading to nine non-rigid tests. Hence, 36 trails of elastic alignments were yielded in total for four 4D CT sequences. We also compared the results adopting NJAD-GD and JAD without considering spatial information. Finally, 72 elastic registration tests were conducted for two methods. In order to quantify the test errors, target registration error (TRE) and Hausdorff distance meansure (HDM) were calculated. HDM is a widely-used measure to calculate the distance of two clouds of points. In the 3D thoracic CT registration, the manually marked landmarks can be applied to calculate HDM of two images.

**Figure 5:**
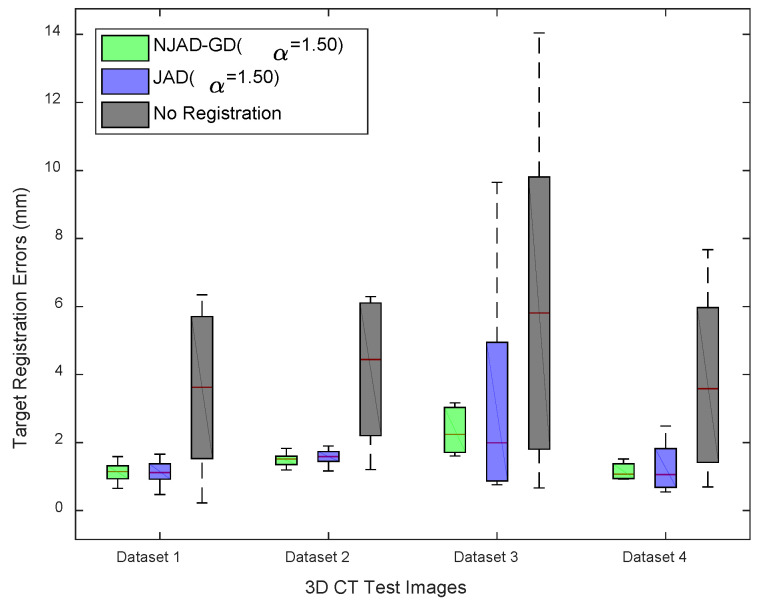
The TREs obtained when employing NJAD-GD algorithm, the registration method based on JAD without gradient distribution.

**Figure 6:**
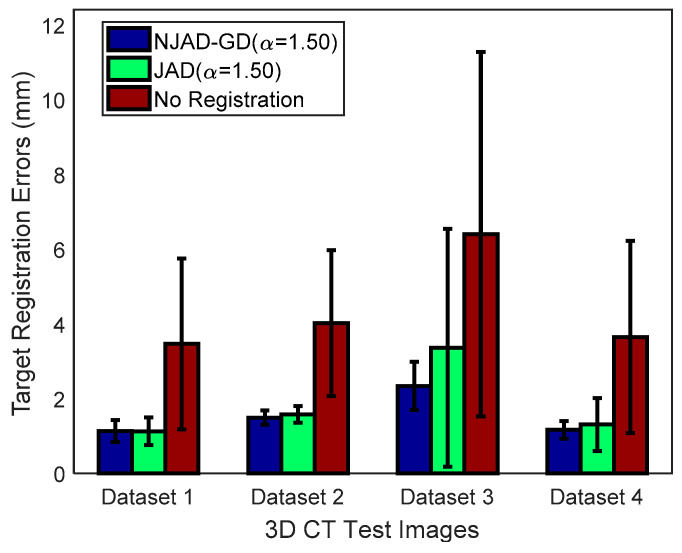
Statistics of TREs before registration and after alignment exploiting the NJAD-GD, JAD methods.

**Figure 7:**
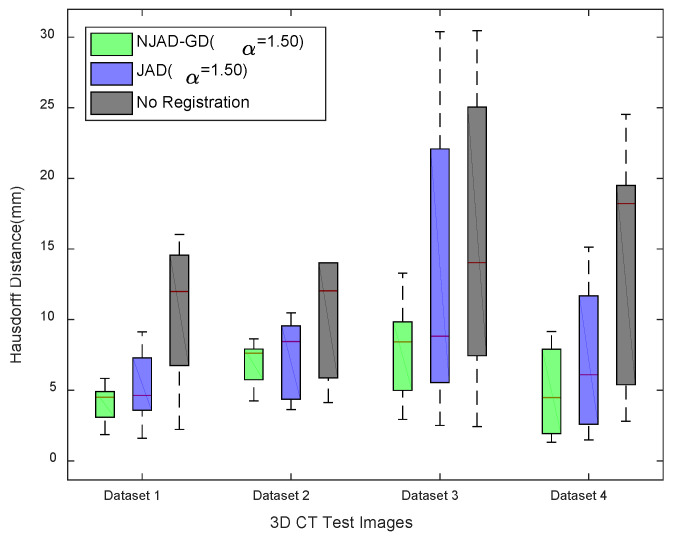
HDMs obtained when employing NJAD-GD algorithm, the registration method based on JAD without gradient distribution.

Figure 5 and Figure 6 demonstrate the registration results of 72 tests, along with TREs before registration and after alignment. Figure 7 illustrates the box-and-whisker plots of HDM values of four 4D CT sequences. It is observed from these results that the registration errors applying NJAD-GD algorithm are less than these obtained by the method based on JAD without gradient distribution.

In implementation of experiments, the nonextensive parameter α = 1.5, bins M = 16, the number of random samples N = 8000, the spacing of mesh points δ = 20 × 20 × 20. The weighting parameters λ_1_ = 0.005 and λ_2_ = 0.001. 

### 4.4. Registration of 3D Cardiac CT Image

**Figure 8:**
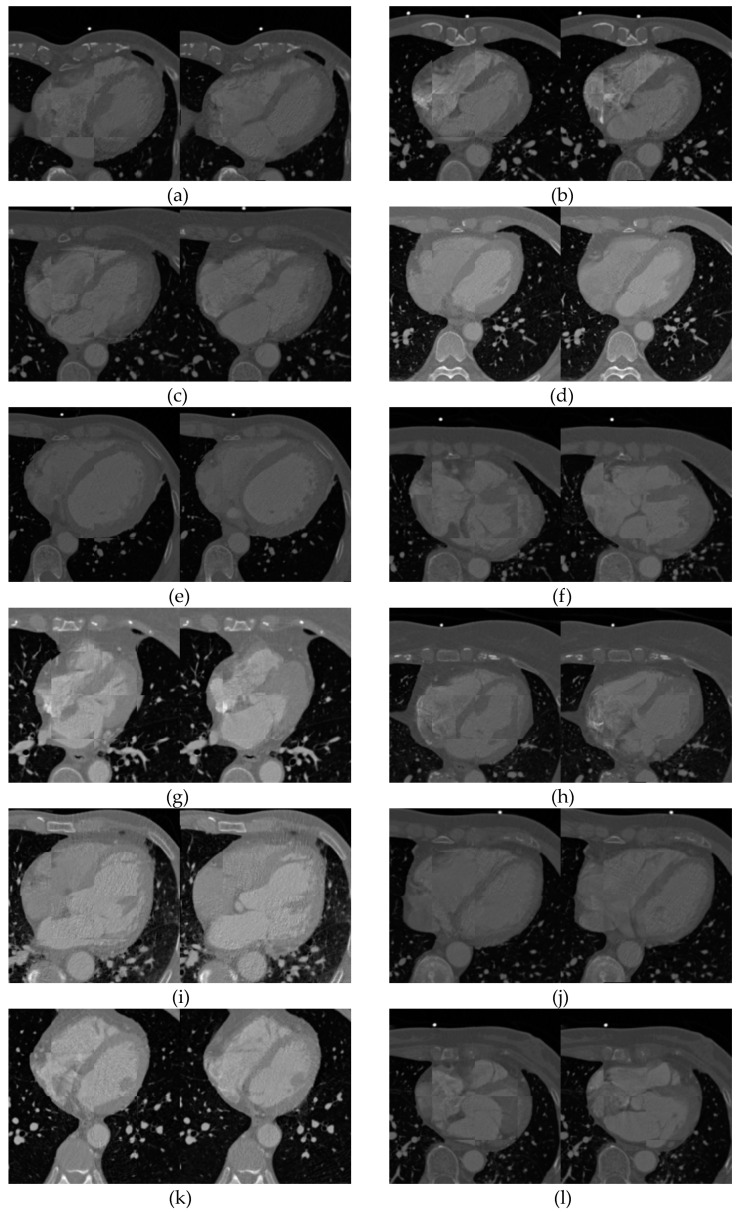
Registration results of 12 groups of 3D cardiac images. (**a**–**l**) display the test results of patient 1 to 12, respectively. In each group, left image represents the checkboard before registration, and the right accounts for the result after registration.

The cardiac CT data consists of 12 groups of 4D image sequence, and each of which includes 10 3D images acquired from one whole cardiac cycle. One cardiac cycle consists of the phase of systole and phase of diastole. In each 4D CT sequence, two 3D images with the maximum deformation were employed as the test images, and 12 nonrigid registration experiments were carried out adopting NJAD-GD approach. Figure 8 illustrates the checkboard of 12 examples adopting our non-rigid framework (α = 1.50) for 12 4D CT sequences. As it can be seen, the registration algorithm based on gradient distribution demonstrates the accuracy results.

In these tests, a multiresolution scheme with three levels was also exploited to implement these nonrigid registrations. Due to the large deformation, the number of random samples N and the spacing of mesh points δ were set to 10,000 and 10 × 10 × 10, with other parameters being as follows: bins M = 16, the weighting parameters λ_1_ = 0.005 and λ_2_ = 0.001, the maximum number of iterations of the limited memory BFGS scheme NMAX = 200.

## 5. Conclusions

In this work, we review the definition and properties of Arimoto entropy, with an information measure based on Arimoto entropy, called JAD. The gradient distributions of reference image and float image are constructed and a distance between them is derived. Additionally, a normalized dissimilarity measure based on JAD was presented. A nonrigid registration method exploiting the normalized measure with gradient distributions is proposed. 

Arimoto entropy is regarded as a generalized form of the classical Shannon entropy. In the aforementioned section, it is proofed that the JAD measure is equal to MI when α tends to 1. We adopted FFDs as the parameter space for non-rigid registration, along with objective function E including three elements: the normalized JAD as the dissimilarity measure, a regularization to acquire the smooth deformation and a distance term of the gradient distributions.
